# Healthy eating perceptions of mothers and caregivers of children in South Africa

**DOI:** 10.4102/hsag.v28i0.2345

**Published:** 2023-09-29

**Authors:** Suzan M. Mokone, Mashudu Manafe, Lindiwe J. Ncube

**Affiliations:** 1 Department of Human Nutrition and Dietetics, School of Health Care Sciences, Sefako Makgatho Health Sciences University, Pretoria, South Africa; 2 Division of Hospitality and Tourism, University of Mpumalanga, South Africa

**Keywords:** perceptions, healthy eating, mothers, caregivers, preschool, children

## Abstract

**Background:**

The perceptions of mothers and caregivers of children attending early childhood development (ECD) centres play a very critical role in promoting healthy eating habits in children and impact long-term health consequences. Food preferences that are developed during childhood continue into adolescence and adulthood and are difficult to change.

**Aim:**

The aim of the study was to assess the perception of mothers and caregivers of children attending ECD centres regarding healthy eating practices.

**Setting:**

The study was conducted in ECD centres in Gauteng, North West and Limpopo.

**Methods:**

A quantitative descriptive design was used to collect data among 290 respondents using a structured, researcher-administered questionnaire.

**Results:**

The findings indicated that the majority (77%) of respondents did not believe that choosing a healthy diet is a matter of knowing foods that are good and bad for health. The majority (59%) of respondents believed that the food they eat and drinks are healthy and see no need to make changes.

**Conclusion:**

The study findings showed that the majority of respondents lack awareness about choosing healthy diets. Furthermore, the current dietary patterns of respondents and their children will remain the same as long as the mothers and caregivers feel that the food they eat is healthy.

**Contribution:**

The findings of the study contribute to the appropriate measures of educating mothers and caregivers in offering healthy diets to children at home and in early childhood centres.

## Introduction

Early childhood is a critical stage for obesity prevention, and early childhood development (ECDs) centres are an ideal setting for healthy eating and nutrition intervention (Skouteris et al. 2020; World Health Organization 2018). However, existing literature indicates that the perception of caregivers and mothers of children attending ECD centres can be a barrier to the successful implementation of healthy eating interventions in this setting (McSweeney et al. 2016). Studies show different results about healthy eating perceptions of children’s mothers and caregivers. According to Lilo, Muñoz and Cruz (2019), the perception of mothers or caregivers on healthy eating is associated with the provision of healthy meals to their children and their feeding practices, including the nutritional status of the children (Mardhiah, Ekayanti & Setiawan 2019; Motebejana, Nesamvuni & Mbhenyane 2022). A South African study by Chakona (2020) reported that the majority of caregivers perceived that eating a variety of foods, including dairy products, vegetables, fruits and low fatty foods, is good for health. However, most caregivers indicated that these foods were expensive and not affordable as they relied on the monthly grant of R410.00 provided by the government (Chakona 2020). Furthermore, according to a Zimbabwean study done by Lewis et al. (2022), the caregivers perceived that introducing a variety of foods in meals will lead to the introduction of some unknown foods and may be unfamiliar to children and other family members.

The perceptions of caregivers regarding healthy eating influence the nutritional quality of meals provided to their children (Tang et al. 2018). For example, a study done by Sharma et al. (2018) indicated that caregivers in Nepal perceived that food such as eggs, meat, milk, fruits and vegetables were healthy, while a study done by Lilo et al. (2019) in the United States reported that most Hispanic caregivers were aware of the importance of eating fruits and vegetables and that these food items contribute to good health as compared to commercial snacks (sweets, biscuits) as well as juicy drinks. However, caregivers did not know how many fruits and vegetable servings are recommended for children’s daily consumption. While a study done by Chakona (2020) reported that caregivers perceived that providing snacks such as Nik Naks daily is convenient and affordable, foods such as noodles had no vitamins and minerals.

Perceptions regarding food intakes may differ in different countries. A United Arab Emirates study done by Ismail et al. (2022) indicated that mothers or caregivers perceived that consuming fruits and vegetables daily is important for maintaining good health; however, they indicated that starchy foods should be consumed less. Differently, a South African study done by Chakona (2020) reported that mothers and caregivers perceived that starchy foods should be consumed in most meals, whereas the Zimbabwean National Survey Report (2018) indicated that the majority of caregivers perceived that healthy diets comprise mainly of vegetables as well as starchy foods.

### Problem statement line

Additionally, Arabian caregivers were aware that consuming foods that are low in fibre can contribute to health problems (Ismail et al. 2022), while a study in the United States done by Fisher et al. (2015) reported that mothers and caregivers did not consider the nutritional value of foods given to their children and families. Furthermore, a South African study done by Besselink et al. (2020) reported that caregivers were knowledgeable about the consequences of eating unhealthy foods. Contrarily, a study done by Okop et al. (2016) reported that participants were not aware that unhealthy eating is associated with chronic lifestyle diseases.

The nutritional knowledge of caregivers influences the dietary intakes and nutritional status of their children (Debela et al. 2017; Hossain et al. 2019). In South Africa, a study conducted at Vhembe district reported that half of caregivers did not know their children’s nutritional needs, which was associated with the consumption of poor diets. Consequently, their children’s nutritional status may be affected (Motadi et al. 2015). In the United States, a study done by Vittrup and McClure (2018) reported that caregivers did not know the adequate food portion sizes for their children, and they offered large portion sizes that contributed to weight gain among children. Caregivers were not aware that their children were overweight; thus, they were not aware of the risk factors of obesity. Similarly, a study done in Indonesia reported that caregivers influence the eating habits of children from an early age by controlling what children eat and the type and number of food items consumed per meal per day (Mardhiah et al. 2019).

Children during the early childhood period depend on mothers and caregivers to provide nutritious meals that promote growth and development. Therefore, the perceptions of mothers and caregivers on healthy eating contribute to the meals provided to their children. The literature discussed earlier shows that caregivers’ and mother’s healthy eating perception is critical to the successful implementation of healthy eating interventions in this setting. Hence, there is a dearth of South African literature on this topic. Therefore, the purpose of the study was to assess the healthy eating perceptions of mothers and caregivers of children attending ECD centres in Gauteng, North West and Limpopo to be able to develop nutrition intervention that promotes healthy eating and improves the quality of life. The study objective was to assess the perceptions of mothers and caregivers of children attending ECD centres regarding healthy eating practices.

## Research methods and design

Using a quantitative descriptive design, data were collected from 290 respondents, mothers and caregivers of children attending ECD centres in three provinces of South Africa, namely, Gauteng (Soshanguve), North West (Moretele) and Limpopo (Makhuduthamaga) to determine their perception of healthy eating. A cluster sampling strategy was used to select ECDs that participated in the study from selected provinces. There are 11 municipality areas in Soshanguve (urban setting), and all ECDs were allocated based on their area of 11 clusters. While in Moretele (rural setting), there are four municipality areas, of which ECDs were allocated based on their area of four clusters, Makhuduthamaga (rural setting) has three municipality areas and ECDs were allocated based on their three clusters. Then the simple random sampling technique was employed by indicating each name of ECDs on a piece of paper, then placing all in the bowl and was shaken and thoroughly mixed. The researcher picked the names of ECDs being blindfolded. Mothers and caregivers from the selected ECDs in each province who met the inclusion criteria were recruited; those who agreed and gave consent to participate in the study formed the sample of the study. In this study, the sample size for the study was calculated from the targeted ECDs registered with the Department of Social Development financial year 2017 or 2018. An online sample size calculator (Raosoft) was used to calculate the sample size, with the population size of 3250 of mothers and caregivers from ECDs, 5% margin of error and a 95% confidence interval. A minimum sample of 285 was calculated and buffered with 5% to make a sample of 290. Out of the sample size of 290, 199 respondents were from Gauteng ECDs, 49 respondents were from North West ECDs and the remaining 47 respondents were from Limpopo ECDs.

The inclusion criteria for this study was the mothers and caregivers who had children in the ECDs registered with the Department of Social Development in 2017 or 2018 financial year and who were willing and gave informed consent to participate in the study. Mothers and caregivers who were absent during data collection days were excluded from the study.

### Data collection

A pilot study was conducted among 29 mothers and caregivers from two ECDs in Soshanguve (10% of study sample size) who met the inclusion criteria. Early childhood development centres chosen for pilot study were not included in the final study. Data collection tool was modified based on the outcome of the pilot study before the actual study was conducted. After ethical clearance for the study was obtained, three months before the data collection process, two research supervisors who guided the research process and the researcher visited the study sites and had information sessions with the principals of ECDs to explain the purpose of the study and provided information to recruit mothers and caregivers to be part of the study. Data were collected over a period of 2 months (June 2019 and July 2019). In Soshanguve, data were collected from 3 June 2019 to 28 June 2019 (weekdays); in Moretele, data were collected from 6 July 2019 to 19 July 2019 (weekdays) and in Makhuduthamaga, data were collected from 21 July 2019 to 30 July 2019 (weekdays). Fieldworkers in the different provinces were trained by the researcher on how to administer the data collection tool. The respondents who met the criteria of the study were selected by using a simple random sampling technique. The consent forms were completed, and private rooms that the ECDs offered were used to collect data. A structured researcher-based questionnaire was used to collect data, and participants entered the interviewing room one at a time. Both the researcher and trained field workers collected data on the perception of healthy eating as well as sociodemographic data of mothers and caregivers of children attending ECDs.

### Data analysis

The completed data from questionnaires were entered in the Excel spreadsheet (cleaned and validated) and imported to the Statistical Package for Social Sciences (SPSS) version 23 for data analysis. Descriptive data (mean, standard deviations and frequency distribution) and inferential statistics (logistic regression) were used to analyse data. A *p*-value was considered statistically significant at *p* < 0.05.

### Ethical considerations

An application for full ethical approval was made to the Sefako Makgatho University Research Ethics Committee (SMUREC) and the consent of the ethics was received in 2019. The ethics approval number is (SMUREC/H/97/2019/PG). All procedures performed in studies involving human participants were following the ethical standards of the institutional and/or national research committee and with the 1964 Helsinki Declaration and its later amendments or comparable ethical standards. Verbal informed consent was obtained from all individual participants involved in the study.

## Results

The sample comprised 290 respondents, of whom 95 (32%) were aged between 20 years and 29 years. The majority (*n* = 158, 54%) of respondents had grade 12 qualifications while 10% had higher education certificates or diplomas and/or degrees. The majority (*n* = 124, 43%) of the respondents were unemployed, and 108 (37%) had their source of income from one person in the family. About 159 (55%) respondents were single. The majority of the respondents (*n* = 112, 39%) reported an income of between R2000.00 and R2999.00 (Table 1).

**TABLE 1:** Sociodemographic information of mothers and caregivers in early childhood developments.

Variables	*n*	%
**Age (years)**		
18–19 years	62	21
20–29 years	95	32
30–39 years	79	27
40–49 years	52	18
Other	2	1
**Education level**		
Grade 1–7	19	7
Grade 8–11	83	29
Grade 12	158	54
Certificate or diploma or degree	30	10
**Employment status**		
Employed	37	13
Self-employed	30	10
Part-time employed	55	19
Unemployed	124	43
Scholar	43	1
**Source of income**		
One person	108	37
Both parents and caregivers	74	26
Social grant	87	30
Other family members	19	7
Other	2	1
**Marital status**		
Single	159	55
Married	96	33
Divorced	6	2
Widowed	15	5
Separated	14	5
**Income (rand)**		
< 1000	28	10
1000–1999	75	26
2000–2999	112	39
3000–3999	40	14
4000–4999	18	6
> 5000	17	6

### Choosing a healthy diet is a matter of knowing good and bad foods

**FIGURE 1:**
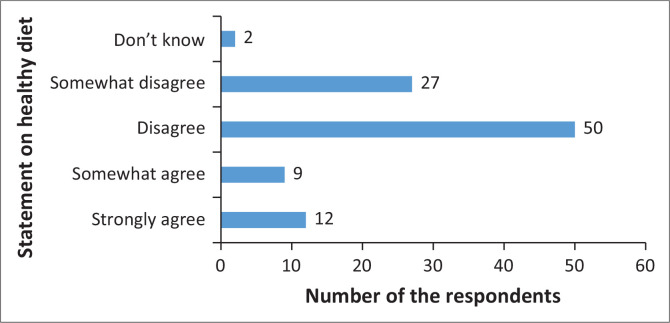
Choosing a healthy diet is a matter of knowing good and bad foods.

As Figure 1 shows, the majority (77%) of the respondents disagree with the statement that says, ‘Choosing a healthy diet is a matter of knowing good and bad foods’.

### Eating a variety of foods each day probably gives you all the vitamins and minerals you need

**FIGURE 2:**
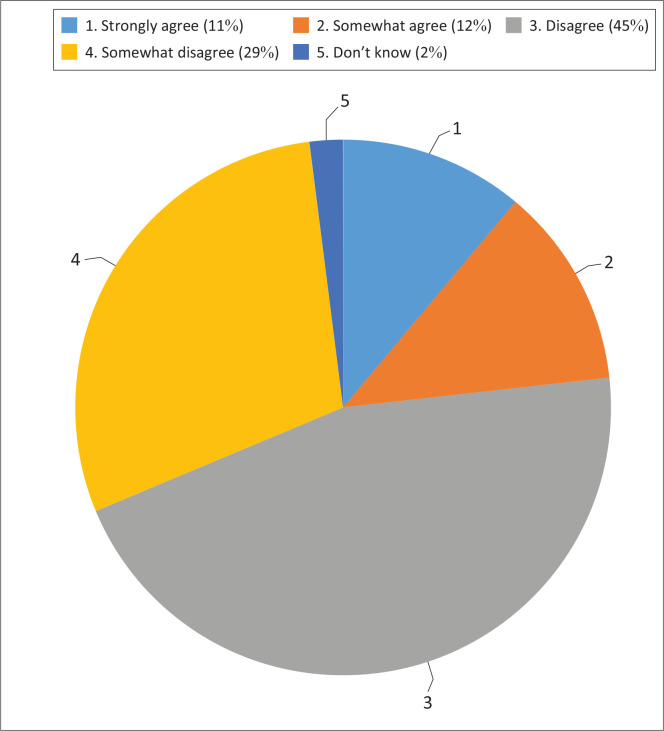
Variety of foods.

The majority (74%) of respondents disagree with the statement that says, ‘Eating a variety of foods each day probably gives you all the vitamins and minerals you need’ (Figure 2).

### Starchy foods like bread, potatoes and rice make people fat

**FIGURE 3:**
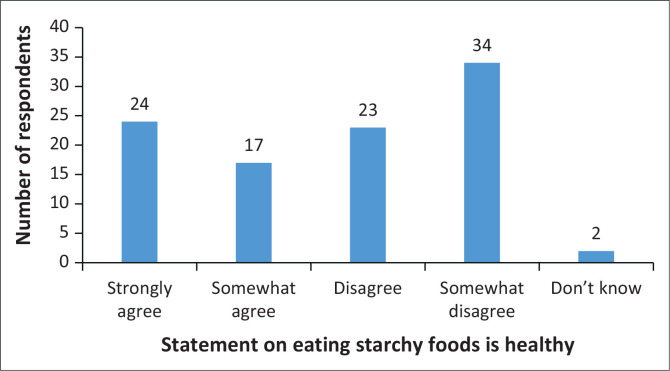
Eating starchy foods.

Most of the respondents (57%) disagree with the statement that says, ‘Starchy foods like bread, potatoes and rice make people fat’, and 31% agree with the statement (Figure 3).

### What you eat can make a big difference at your chances of getting some diseases like heart disease or cancer

**FIGURE 4:**
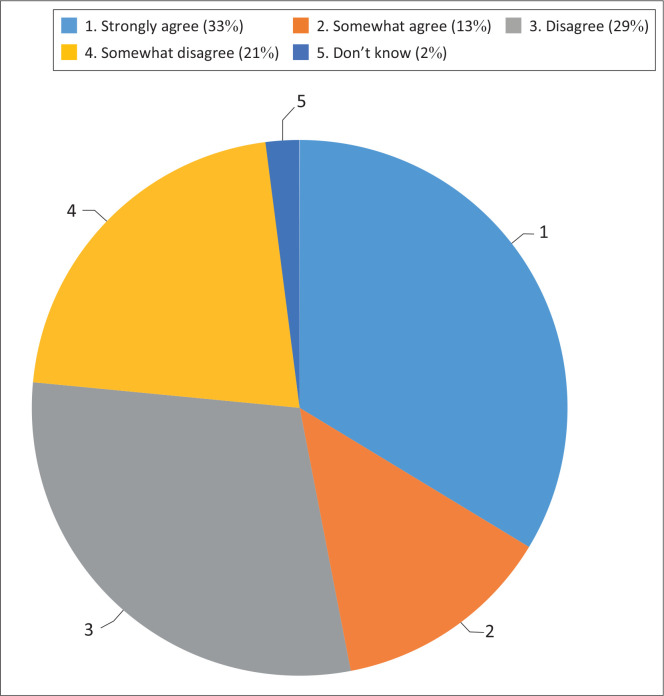
What you eat makes a big difference.

As indicated by Figure 4 most of the respondents (50%) disagree with the statement that says, ‘What you eat can make a big difference at your chances of getting diseases like heart disease or cancer’, and 46% agree with the statement.

### The things I eat and drink are healthy, so there is no reason for me to make changes

**FIGURE 5:**
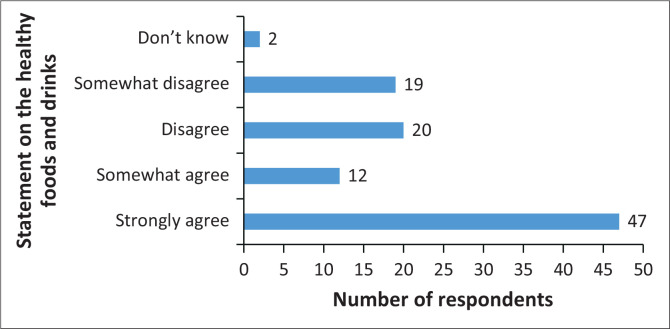
Food eaten and drinks are healthy.

Figure 5 shows that 59% of the respondents agree with the statement that says, ‘The things I eat and drink are healthy, so there is no reason for me to make changes’, and 39% do not agree with the statement.

### Summary of logistic regression between variables

The findings of the study indicate that the income of the respondents was associated with an individual making changes to their diet by a factor of 1.30. The level of respondents’ education was associated with the belief of what individuals eat make a difference at their chances of getting diseases by a factor of 1.56 as indicated in Table 2.

**TABLE 2:** Logistic regression analysis.

Variables	Odds ratio	95% CI	*p*
**Choosing a healthy diet is a matter of knowing good and bad foods**			
Age	1.20	0.79–1.83	0.37
Marital status	0.92	0.61–1.37	0.69
Income	2.55	1.59–4.08	0.00*
Education	2.23	1.15–4.33	0.01*
**Eating a variety of foods each day probably gives you all the vitamins and minerals you need**			
Age	0.91	0.58–1.41	0.68
Marital status	0.94	0.62–1.42	0.79
Income	3.11	1.89–5.10	0.00*
Education	1.81	0.92–3.55	0.08
**Starchy foods like bread, potatoes and rice make people fat**			
Age	0.86	0.62–1.19	0.36
Marital status	1.48	1.03–2.11	0.03*
Income	1.36	1.03–1.81	0.02*
Education	1.73	1.10–2.73	0.01*
**Many recommendations about healthy ways of eating**			
Age	0.65	0.47–0.90	0.00*
Marital status	1.12	0.85–1.48	0.39
Income	1.45	1.10–1.90	0.00*
Education	1.99	1.28–3.09	0.00*
**What you eat can make a big difference at your chances of getting a diseases like heart disease or cancer**			
Age	1.004	0.75–1.33	0.94
Marital status	1.02	0.79–1.30	0.86
Income	1.25	0.99–1.59	0.06
Education	1.56	1.05–2.30	0.02*
**The things I eat and drink are healthy, so there is no reason for me to make changes**			
Age	1.11	0.84–1.46	0.45
Marital status	1.24	0.98–1.59	0.07
Income	1.30	1.04–1.62	0.02*
Education	1.29	0.90–1.85	0.15

CI, confidence interval.

* *p*-value < 0.05.

### Reliability

In this study, reliability was achieved by making use of the standardised data collection tool that was adapted from the study done by Kirsten et al. (2013) in determining the healthy eating perceptions of mothers and caregivers.

### Validity

Construct validity was achieved by training the researcher and research assistants by the two supervisors on how to conduct interviews according to the questionnaire before data collection period. Data were collected according to the structured researcher-based questionnaire and the recording of responses was completed, yielded consistent results during data collection period. Simple random sampling was done and ensured a representative sample from the population.

## Discussion

The study aimed to assess the perceptions of mothers and caregivers of children attending ECD centres in South Africa regarding healthy eating practices. Most of the respondents were between the ages of 20 years and 29 years, unemployed, single and with an income of less than R3000.00. Most of the respondents in this study were mature in age; therefore, perceptions on healthy eating and variety of foods might differ as most might be exposed to education on healthy eating during clinic visits or through the media in their respective provinces. However, this was in line with a study done by Kristo, Angelos and Uzun (2021), which reported that a study done on maternal age is associated with the perceptions on healthy eating. As the majority of respondents are unemployed and have a lower income, they might perceive that healthy food are expensive and then resort to buying cheaper food items that are unhealthy, which might predispose their children to poor nutritional status that can affect growth and development. The findings are similar to a study done in Pakistan by Ahmad, Afzal and Imtiaz (2020), which reported that lower income caregivers perceived healthy eating as expensive and were unable to afford and provide healthy foods for their children. However, this was in contrast to a study done by Kano et al. (2021), which reported that mothers and caregivers who are employed do not have sufficient time to provide healthy meals for the children; instead they resort to junk food with high energy content.

The findings of the study show that many respondents disagree with the statement that choosing a healthy diet is a matter of knowing good and bad foods. These findings are of concern because the perception of healthy eating of caregivers is critical in influencing children to adopt healthy eating, as caregivers are responsible for purchasing and cooking foods (Dumas et al. 2020; Sirasa et al. 2020). The negative perception of healthy eating of caregivers is associated with unhealthy eating practices of children and families (Paes, Ong & Lakshman 2015). Even though caregivers perceived that eating healthy food is important, their dietary intake of some nutrients was inadequate (Kano et al. 2021). The findings showed that choosing a healthy diet was associated with income by a factor of 2.55 (95% CI [1.59–4.08], *p* < 0.05) and education by a factor of 2.23 (95% CI [1.15–4.33], *p* < 0.05). This was similar to the findings that some studies reported that income and education have an association with the provision of healthy eating among caregivers of children (Ahmad et al. 2020; Sirasa et al. 2020). In this study, the income of the respondents was associated with eating a variety of foods by a factor of 3.11 (95% CI [1.89–5.10], *p* < 0.05). Thus, the respondents’ food purchases might be affected as the majority are unemployed and cannot purchase a variety of foods.

Furthermore, most respondents did not believe that eating a variety of foods each day probably gives you all the vitamins and minerals you need. A South African study done in Limpopo by Motebejana et al. (2022) reported that the majority of caregivers did not know the importance of providing a variety of foods to children, which can lead to the prevalence of malnutrition. In Bangladesh, a study done by Hossain et al. (2019) reported that the majority of caregivers had a low perception of the importance of eating a variety of foods. However, this was in contrast to an Arabian study done by Ismail et al. (2022), which reported that caregivers were knowledgeable about the importance of consuming fruits and vegetables daily, which is good for health. However, this knowledge may not necessarily translate to positive behaviour of offering children a variety of foods that would provide vitamins and minerals. Mothers and caregivers are responsible for providing a variety of foods to children and teaching children about the importance of food groups and a balanced diet (Hughes et al. 2019). A study done in Indonesia by Mardhiah et al. (2019) reported that the higher perception of healthy eating of ECD caregivers was associated with a high intake of vegetables and fruits by children. Different factors may play a role in the high intake of fruits and vegetables, which were not investigated in the current study.

The findings of the study indicated that most of the respondents disagreed that starchy foods like bread, potatoes, and rice make people fat. According to the Korean study done by Tang et al. (2018), caregivers indicated that a high intake of foods as well as restricting food is associated with gaining weight and obesity. While an Ethiopian study done by Gebru et al. (2022) reported that caregivers linked unhealthy eating to being bad for their health. In South Africa, a study done by Strydom, Du Plessis and Daniels (2021) reported that caregivers indicated that consuming too many starchy foods in a meal can lead to gaining weight. However, the responses were not based on the quantity of starch consumed.

The findings of the study indicated that 47% of the respondents believe that foods that individual eats can make a big difference at their chances of getting diseases like heart disease or cancer. However, this might not be a practiced behaviour. As Yaseen et al. (2020) reported in their study, the majority of caregivers perceived that food intake is not linked to health outcomes. In Ethiopia, a study done by Berhane et al. (2018) reported that caregivers were aware that consuming unhealthy foods affects health and contributes to the development of some diseases. Similarly, in China, according to the study done by Shao et al. (2022), caregivers were aware that poor diets can lead to overweight and obesity, while in Bangladesh, a study done by Rahman et al. (2016) reported that caregivers perceived that healthy foods do not taste as good as unhealthy foods. The Arabian study done by Ismail et al. (2022) reported that caregivers indicated that foods that are low in fibre can cause health issues and can lead to diseases. An Ethiopian study done by Gebru et al. (2022) and a Nepal study done by Sharma et al. (2018) reported that caregivers perceived that healthy eating has benefits of contributing to good health and improved long-term consequences. Education level in the study was associated with the belief that what individuals eat can make a difference in their chances of getting diseases by a factor of 1.56 (95% CI [1.05–2.30], *p* < 0.05). Most of the respondents had secondary education and can thereby have access to materials that encourage healthy eating. The education levels of mothers and caregivers enable them to read printed material on childhood nutrition guidelines and recommendations and were knowledgeable about healthy eating. This was in line with another South African study done by Madiba, Chelule and Mokgatle (2019), and an Ethiopian study done by Dessie et al. (2019) reported that higher maternal education was associated with better nutritional status of preschool children because of higher income and provision of a variety of foods.

The findings of the study indicated that only 47% of the respondents are of the view that what they eat and drink are healthy and find no reason to make changes. This was in line with an Australian study done by Khokhar et al. (2019), which reported that the majority of caregivers were concerned about the amount of fat, sugar and salt added to their meals and perceived that reducing the amount of salt, fats and sugar in their meals will lead to better health. In a study by Sharma et al. (2018), caregivers reported that they preferred unhealthy junk foods such as snacks and beverages because of being convenient as compared to healthy foods that take time to prepare. While a South African study done by Strydom et al. (2021) reported that because of the high unemployment rate, caregivers are most likely to encounter a challenge in providing recommended healthy foods that are costly. The findings of the study showed that income was associated with an individual making changes to their diet by a factor of 1.30 (95% CI [1.04–51.62], *p* < 0.05). This might resonate with the respondents in the study as most of them were unemployed and may not afford to buy healthy food.

### Strengths and limitations

The strengths of the study include the following:

- Validated data collection tools were used for data collection
- The sample included mothers and caregivers from three different provinces, representing both urban and rural settings.

**The study has some limitations:** only mothers and caregivers from ECDs registered with the Department of Social Development were included in the study, and private ECDs and nonregistered ECDs were excluded.

### Recommendations

The findings of the study give insight into the perceptions of mothers and caregivers on the importance of healthy eating and the provision of a variety of foods; this will assist in developing an intervention for mothers and caregivers that will raise awareness of the importance of healthy eating and promotion of a variety of foods that will improve health.

## Conclusion

The findings of the study indicate that the majority of mothers and caregivers did not perceive that choosing a healthy diet is a matter of knowing which foods are good and bad for health and that eating a variety of foods gives you all the vitamins and minerals the body needs. Therefore, the study recommends an intervention that will raise awareness of the importance of healthy eating among mothers and caregivers and the benefits of providing a variety of foods to improve health. Further studies are necessary to be conducted in other South African provinces including private ECDs to assess the perceptions and practices among caregivers, mothers and children.
